# Prognostic impact of effusion in multiple body cavities after allogeneic hematopoietic stem cell transplantation

**DOI:** 10.1007/s12185-025-03949-7

**Published:** 2025-03-03

**Authors:** Yasutaka Masuda, Akira Honda, Takashi Oyama, Yosuke Masamoto, Mineo Kurokawa

**Affiliations:** 1 Department of Hematology and Oncology, Graduate School of Medicine, The University of Tokyo, 7-3-1 Hongo, Bunkyo-Ku, Tokyo, 113-8655 Japan; 2 Department of Cell Therapy and Transplantation Medicine, The University of Tokyo Hospital, 7-3-1 Hongo, Bunkyo-Ku, Tokyo, 113-8655 Japan

**Keywords:** Fluid retention, Cavity effusion, Allogeneic hematopoietic stem cell transplant

## Abstract

Fluid retention presenting as effusions in body cavities is sometimes encountered following allogeneic stem cell transplantation (allo-HSCT). It is unclear whether cavity effusions at independent sites may serve as cumulative correlates of fluid overload and whether a higher number of effusion sites are associated with a worse prognosis. Here, we comprehensively reviewed pleural, peritoneal, and pericardial effusions in 178 first allo-HSCT recipients retrospectively. A total of 123 (69.1%) patients developed effusions in any cavity. New pleural, peritoneal, and pericardial effusions were found after allo-HSCT in 106, 88, and 53 patients, at a median of 38.0 (range, 2–2950), 22.5 (range, 2–1324), and 40 (range, 2–945) days, respectively. The cumulative incidence at day 100 was 41.0%, 40.4%, and 20.8%, respectively. Of the 92 patients who presented with effusions by day 100, 28 patients presented with effusion in a single cavity, 39 in two cavities, and 25 in all three cavities. The 2-year overall survival rates of patients with effusions in zero, one, two, and three cavities by day 100 were 86.1%, 60.0%, 59.6%, and 18.8%, respectively, showing an additive adverse association with outcome. Prospective studies to further characterize fluid dynamics following allo-HSCT are warranted.

**Supplementary Information:**

The online version contains supplementary material available at 10.1007/s12185-025-03949-7.

## Introduction

The outcome of allogeneic hematopoietic stem cell transplantation (allo-HSCT) has improved over the last decades [1–3]; however, a unique array of complications still puts some patients at risk for potentially life-threatening situations. Thus, comprehensive assessment of the patient’s status and timely management are warranted to improve transplantation outcomes. Fluid retention presenting as effusions in body cavities is sometimes encountered post-allo-HSCT. Few studies have examined cavity effusions in adult patients undergoing allo-HSCT. While the causal link between effusions and prognosis is still obscure, previous studies have suggested that cavity effusions could be surrogates for poor survival [4–8]. These studies examined pleural (PL), peritoneal (PT), or pericardial (PC) effusions independently, and few studies have addressed when and where cavity effusions in different locations occur and how much those are related to prognosis. Under the hypothesis that cavity effusions at independent sites may serve as cumulative correlates of fluid overload and may be associated with a worse prognosis depending on the number of effusion sites, we conducted a comprehensive analysis of effusions in three body cavities simultaneously post-allo-HSCT to identify predictive factors for and prognostic impact of effusions.

## Methods

### Patients and data collection

We conducted a retrospective study of adult patients (aged ≥ 16) who underwent their first allo-HSCT between January 2010 and December 2020 at the University of Tokyo Hospital. Patients who underwent computed tomography (CT) allowing cavity effusions at least once after transplantation were included for analysis. The clinical data were collected from electronic medical records. This study was approved by the Ethics Committee of the University of Tokyo Hospital.

The size of PL effusion was classified into small (≤20% of the hemithorax), moderate (20–40%), and large (>40%) based on the estimation of the anteroposterior quartile [9]. The size of PT effusion was classified into small when confined to the rectovesical pouch and large in other cases [6]. PC effusion was defined as a fluid collection within pericardium with a maximal depth of 10 mm or more [7].

### Definitions

For malignant hematological diseases, refined disease risk index (DRI) was defined as previously described [10]. For non-malignant and non-hematological diseases, DRI was defined low. For solid tumors, DRI was not defined. Myeloablative conditioning was defined as regimens that included either total body irradiation >8 Gy, busulfan >9 mg/kg, or melphalan >140 mg/m^2^, and all other regimens were considered reduced intensity conditioning [11, 12]. The donor group was classified according to the number of mismatched alleles of human leucocyte antigen (HLA) loci (HLA-A, -B, -C, and -DR) for the graft-versus-host direction. The Hematopoietic Cell Transplantation-Comorbidity Index was evaluated based on the criteria by Sorror et al. [13]. Neutrophil engraftment was defined as the first day of three consecutive days with absolute neutrophil count exceeding 0.5 × 10^9^/L. Performance status was evaluated as per Eastern Cooperative Oncology Group Performance Status Scale [14]. Acute GVHD, thrombotic microangiopathy (TMA), and sinusoidal obstruction syndrome (SOS) were diagnosed according to the traditional criteria [15–20]. Cytomegalovirus (CMV) viremia was defined as three or more positive cells per two slides (CMV pp65 antigenemia C10/C11) [21]. Pre-transplant EASIX was calculated by lactate dehydrogenase (U/L) × creatinine (mg/dL)/thrombocytes (nL), each obtained before conditioning chemotherapy [22].

During the first 100 days post-allo-HSCT (D100) and at the time of effusion recurrence, the number of concurrent cavities with effusion (zero to three) was counted. In a patient who underwent several CT examinations by D100, the maximum number of effusion-positive cavities was used for analysis. For the analysis of the prognosis according to the attenuation of effusions, the date when the number of affected cavities reached the maximum by D100 was defined as the date of onset of effusion.

### Endpoints

The primary endpoint was the 2-year overall survival (OS, time from transplantation until death from any cause) rate. Secondary endpoints included 100-day and 2-year cumulative incidence of cavity effusions, the 2-year cumulative incidence of relapse (CIR, the first documented relapse or progression of the disease) rate, the 2-year non-relapse mortality (NRM, death without evidence of relapse or progression of the disease) rate, and the relation between patient characteristics and the onset of cavity effusions.

Patients who were alive at the last follow-up or who received a second transplantation were censored. Disease relapse was diagnosed with molecular, morphological, and/or imaging findings. When complete response was not achieved after transplantation in cases with a pre-transplantation disease status of not in remission in malignant disorders, the day of relapse was defined as day +0.1 from the landmark point (described below).

### Statistical analysis

Pearson’s chi-squared test was used for comparing categorical data. Wilcoxon signed-rank sum test and Mann–Whitney *U* test were used for comparing continuous data with paired and unpaired variables, respectively. The probability of OS was estimated according to the Kaplan–Meier method, and the groups were compared using the log-rank test; Holm–Bonferroni correction was applied for the comparisons among more than two groups. Probabilities of CIR, NRM, and cavity effusions were analyzed by cumulative incidence methods, and the groups were compared using the Gray test [23]. Relapse and NRM were considered competing risks with each other. The competing risk for cavity effusions was death before cavity effusions of interest. The Cox proportional hazard regression model was employed to identify the factors influencing OS, and the Fine–Gray competing risk regression model for CIR, NRM, and cavity effusions. Wald test was employed to assess overall *P* values for factors with >2 levels (e.g., number of cavities of effusion). Univariate and multivariate analyses were performed to identify predictive factors for cavity effusions after allo-HSCT. Variables with a *P* value <0.1 in the univariate analysis were subjected to the multivariate analysis.

The effects of clinical events occurring post-transplantation on clinical outcomes were analyzed among patients who survived without undergoing second HSCT by D100 (landmark point).

All tests were two sided, and *P* < 0.05 was considered statistically significant. Statistical analyses were performed with R (version 4.4.1; https://cran.r-project.org) and EZR (Saitama Medical Center, Jichi Medical University, Saitama, Japan) [24], which is a graphical user interface for R version 4.3-1 (R Foundation for Statistical Computing, Vienna, Austria). Figure presentation was performed either with EZR or with Python software program for Mac (version 3.8.18; https://www.python.org).

## Results

### Patient characteristics

During the study period, 191 patients underwent the first allo-HSCT at our department. 178 patients who underwent CT examinations at least once post-allo-HSCT were subjected to further analysis. Table 1 summarizes the baseline characteristics of the studied population. Post-transplant cyclophosphamide was not used in any patients.

**Table 1:** Characteristics of studied patients

Characteristics	Value
Age—no. (%)	
<50	93 (52.2)
≥50	85 (47.8)
Recipient sex—no. (%)	
Male	111 (62.4)
Female	67 (37.6)
Sex match between recipient and donor—no. (%)	
Match	103 (57.9)
Male to female	28 (15.7)
Female to male	39 (21.9)
Not confirmed	8 (4.5)
Diagnosis—no. (%)	
AML	69 (38.8)
Other myeloid neoplasms^a^	34 (19.1)
Lymphoid and ambiguous lineage neoplasms^b^	57 (32.0)
Inborn errors of metabolism^c^	14 (7.9)
Others^d^	4 (2.2)
Refined DRI—no. (%)	
Low/intermediate	104 (58.4)
High/very high	71 (39.9)
Not confirmed	3 (1.7)
Donor type—no. (%)	
MRD/MUD	101 (56.7)
MMRD/MMUD	44 (24.7)
CB	33 (18.5)
Cell source—no. (%)	
PBSC	29 (16.3)
BM	116 (65.2)
CB	33 (18.5)
ABO—no. (%)	
Match	74 (41.6)
Major mismatch	35 (19.7)
Minor mismatch	40 (22.5)
Bidirectional mismatch	29 (16.3)
CMV antibody—no. (%)	
Both negative	25 (14.0)
Either positive	133 (74.7)
Not confirmed	20 (11.2)
HCT-CI score—no. (%)	
0	103 (57.9)
≥1	75 (42.1)
ECOG PS—no. (%)	
0	103 (57.9)
≥1	75 (42.1)
Conditioning regimen intensity—no. (%)	
MAC	109 (61.2)
RIC	69 (38.8)
GVHD prophylaxis—no. (%)	
CNI + MTX	160 (89.9)
Others	18 (10.1)
Engraftment day—no. (%)	
<Day 18	43 (24.2)
≥Day 18	121 (68.0)
Not engrafted/not confirmed	14 (7.9)
Grade 2–4 acute GVHD—no. (%)	
No	95 (53.4)
Yes	77 (43.3)
Not confirmed	6 (3.4)
TMA—no. (%)	
No	166 (93.3)
Yes	12 (6.7)
SOS—no. (%)	
No	171 (96.1)
Yes	7 (3.9)
CMV viremia—no. (%)	
No	100 (56.2)
Yes	75 (42.1)
Not confirmed	3 (1.7)

*AML* acute myeloid leukemia, *BMT* bone marrow transplantation, *CB* cord blood, *CBT* cord blood transplantation, *CMV* cytomegalovirus, *DRI* disease risk index, *ECOG PS* Eastern Cooperative Oncology Group Performance Status Scale, *GVHD* graft-versus-host disease, *HCT-CI* Hematopoietic Cell Transplantation-Comorbidity Index, *MAC* myeloablative conditioning, *MMRD* HLA-mismatched related donor, *MMUD* HLA-mismatched unrelated donor, *MRD* HLA-matched related donor, *MUD* HLA-matched unrelated donor, *PBSCT* peripheral blood stem cell transplantation, *RIC* reduced intensity conditioning, *SOS* sinusoidal obstruction syndrome, *TMA* thrombotic microangiopathy

^a^20 patients with myelodysplastic syndrome, 11 with myeloproliferative neoplasms, and 3 with myelodysplastic/myeloproliferative neoplasms

^b^28 patients with acute lymphoblastic leukemia, 26 with other T-cell/NK-cell neoplasms, and 3 with other B-cell neoplasms

^c^14 patients with adrenoleukodystrophy

^d^1 patient with aplastic anemia, 2 with hemophagocytic lymphohistiocytosis, and 1 with invasive thymoma

### Timing of cavity effusions

A total of 123 (69.1%) presented with effusions either in the PL, PT, or PC cavity post-allo-HSCT during the whole observation period. CT examinations were performed with the median of 9 (range, 1–50) times and 5 (range, 1–18) times in patients with and without effusion in at least one cavity, respectively (*P* < 0.001).

**Fig. 1:**
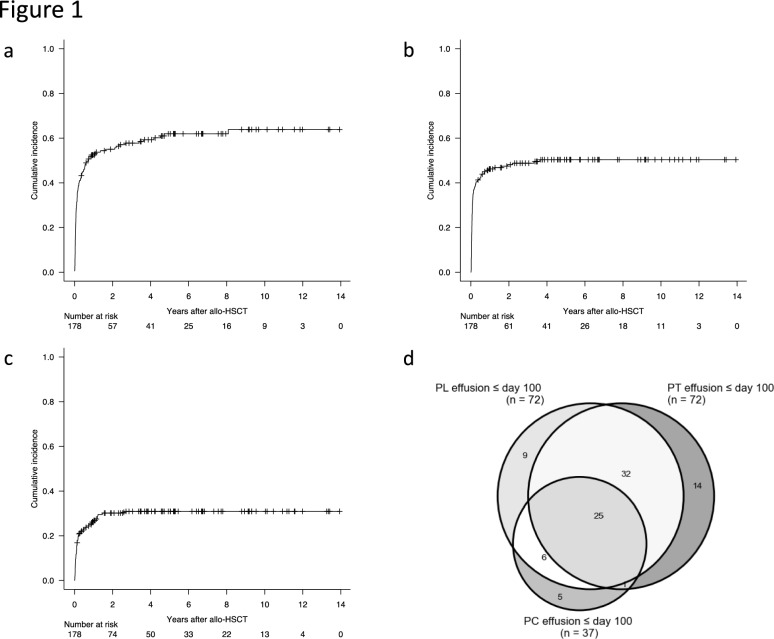
Incidence and locations of cavity effusions following allo-HSCT. Cumulative incidence of pleural (**a**), peritoneal (**b**), and pericardial (**c**) effusions after allo-HSCT. **d** Combinations of effusion locations that presented within 100 days following allo-HSCT

To explore the temporal characteristics of cavity effusions post-allo-HSCT, the date of first or recurrent effusion was studied. The PL, PT, and PC effusions were found in 106, 88, and 53 patients for the first time post-allo-HSCT with a median of 38.0 (range, 2–2950), 22.5 (range, 2–1324), and 40 (range, 2–945) days. The cumulative incidence of PL, PT, and PC effusions were 41.0% (95% confidence interval (CI), 33.7–48.1%), 40.4% (95% CI, 33.2–47.6%), and 20.8% (95% CI, 15.2–27.1%) at D100, and 55.0% (95% CI, 47.3–62.1%), 48.1% (95% CI, 40.5–55.3%), and 30.1% (95% CI, 23.4–37.1%) at 2 years post-allo-HSCT, respectively (Fig. 1a–c).

We next focused on cavity effusions detected by D100. CT examinations were performed with the median of 2 (range, 0–16) times and 4 (range, 1–16) times in patients with and without effusion in at least one cavity by D100, respectively (*P* < 0.001). A total of 92 (51.7%) out of 178 patients presented with effusions in either cavity. The combinations of effusion-positive cavities are presented in Fig. 1d. The PL, PT, and PC were found in 72, 72, and 37 patients, respectively. While 28 (30.4%) out of 92 patients with at least one effusion-positive cavity presented with an effusion in a single cavity, other patients presented with effusions in two or more studied cavities simultaneously or sequentially: 39 (42.4%) patients presented with effusions in two cavities, and 25 (27.2%) in all of three cavities.

Out of 92 patients who presented with cavity effusions by D100, 42 (45.7%) patients developed recurrent cavity effusions at the same cavity beyond D100. The day of recurrence clustered around day 200, with a median of 213 (inter-quartile range, 154–379.3). Effusions in other cavities besides the recurrent cavities were observed in a subset of patients, with the number of affected cavities at recurrence totaling one in 18 patients, two in 17, and three in 7. Most of the effusion relapse occurred at PL: a total of 38, 24, and 11 patients presented with PL, PT, and PC effusions, respectively.

These results show that the majority of the cavity effusions, mostly involving PL and PT cavities, occur acutely within D100 following allo-HSCT, with a subset of patients presenting recurrence beyond D100.

### Prognostic impact of cavity effusions

The median observation period was 778 (range, 8–5088) days, 430 (range, 11–4839) days, and 1413 (range, 8–5088) days in patients with and without effusion in at least one cavity (*P* < 0.001). By D100, 5 patients underwent second allo-HSCT, and 18 died, with 155 remaining alive on D100 (landmark point). As a primary endpoint, the 2-year OS rate of the whole studied population was 63.7% (95% CI, 55.7–70.5%). The 2-year CIR and NRM rate were 30.1% (95% CI, 23.4–37.1%) and 19.7% (95% CI, 14.1–26.0%), respectively.

**Fig. 2:**
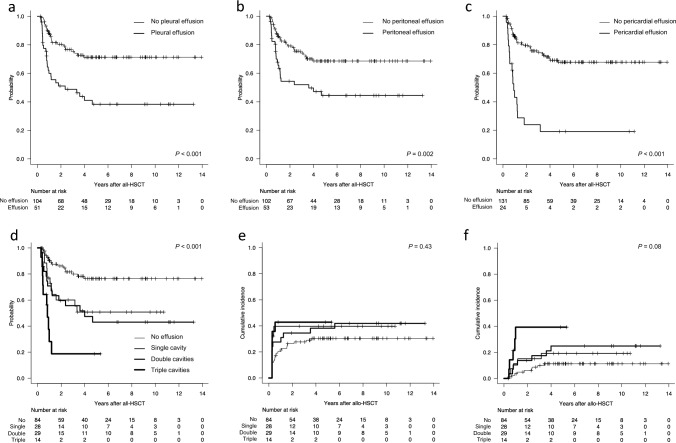
Prognostic impact of cavity effusions following allo-HSCT. Overall survival of patients with pleural (**a**), peritoneal (**b**), and pericardial (**c**) effusions within the first 100 days following allo-HSCT. Overall survival (**d**), cumulative incidence (**e**), and non-relapse mortality (**f**) stratified according to the number of effusion-positive cavities within the first 100 days following allo-HSCT

Our primary goal of the research was to investigate the association of fluid retention in various body cavities and their prognostic impact. On univariate and multivariate analysis to assess the impact of transplantation-related factors on OS, cavity effusions by D100 were associated with significant inferior OS regardless of its locations (Table 2). The presence of effusions was associated with significantly worse OS on univariate analysis. Concomitantly, the 2-year OS rate of patients with PL, PT, and PC effusions was 51.1% (95% CI, 36.0–64.3%), 54.4% (95% CI, 39.2–67.3%), and 23.9% (95% CI, 8.8–43.0%), respectively (Fig. 2a–c).

**Table 2:** Risk factors affecting overall survival, cumulative incidence of relapse, and non-relapse mortality

Characteristics	Overall survival				Cumulative incidence of relapse				Non-relapse mortality			
	Univariate analysis		Multivariate analysis		Univariate analysis		Multivariate analysis		Univariate analysis		Multivariate analysis	
	HR (95% CI)	*P* values	HR (95% CI)	*P* values	HR (95% CI)	*P* values	HR (95% CI)	*P* values	HR (95% CI)	*P* values	HR (95% CI)	*P* values
Age—no. (%)												
<50	1		1		1				1		1	
≥50	1.99 (1.24–3.19)	**0.004**	1.33 (0.61–2.90)	0.47	1.05 (0.64–1.71)	0.85			2.55 (1.35–4.84)	**0.004**	3.49 (1.26–9.64)	**0.02**
Recipient sex—no. (%)												
Male	1				1		1		1			
Female	1.47 (0.92–2.34)	0.10			1.52 (0.93–2.49)	0.10	1.45 (0.89–2.37)	0.14	1.10 (0.60–2.04)	0.76		
Sex match between recipient and donor—no. (%)												
Match	1				1				1			
Male to female	1.62 (0.91–2.90)	0.10			1.22 (0.67–2.24)	0.51			1.50 (0.71–3.20)	0.29		
Female to male	0.89 (0.48–1.64)	0.71			0.67 (0.34–1.34)	0.26			0.83 (0.39–1.78)	0.63		
Diagnosis—no. (%)												
AML	1		1		1		1		1			
Other myeloid neoplasms	1.01 (0.55–1.86)	1.00	0.70 (0.33–1.49)	0.35	0.68 (0.33–1.38)	0.28	0.56 (0.25–1.24)	0.15	1.62 (0.83–3.15)	0.16		
Lymphoid and ambiguous lineage neoplasms	0.85 (0.49–1.48)	0.57	0.68 (0.32–1.47)	0.33	1.34 (0.81–2.21)	0.25	1.32 (0.76–2.27)	0.32	0.85 (0.43–1.67)	0.64		
Inborn errors of metabolism	0.23 (0.05–0.95)	**0.04**	0.57 (0.12–2.74)	0.49	0.00 (0.00–0.00)	**<0.001**	0.00 (0.00–0.00)	**<0.001**	0.52 (0.14–1.98)	0.34		
Others	0.97 (0.23–4.06)	0.96	0.00 (0.00-Inf)	1.00	0.75 (0.10–5.39)	0.77	0.00 (0.00–0.00)	**<0.001**	1.14 (0.13–10.23)	0.91		
Refined DRI—no. (%)												
Low/intermediate	1		1		1		1		1		1	
High/very high	3.32 (2.05–5.37)	**<0.001**	2.39 (1.24–4.60)	**0.009**	2.12 (1.29–3.49)	**0.003**	2.46 (1.48–4.08)	**<0.001**	2.16 (1.17–3.98)	**0.01**	0.74 (0.32–1.71)	0.48
Donor type—no. (%)												
MRD/MUD	1				1				1			
MMRD/MMUD	1.02 (0.59–1.76)	0.94			0.87 (0.48–1.56)	0.63			1.18 (0.62–2.27)	0.61		
CB	1.05 (0.55–2.00)	0.88			1.48 (0.84–2.62)	0.18			1.00 (0.43–2.32)	0.99		
Cell source—no. (%)												
PBSC	1				1				1			
BM	1.57 (0.77–3.18)	0.21			0.68 (0.41–1.11)	0.12			1.29 (0.66–2.55)	0.46		
CB	1.51 (0.63–3.58)	0.35			1.48 (0.84–2.62)	0.18			1.00 (0.43–2.32)	0.99		
ABO—no. (%)												
Match	1				1		1		1		1	
Major mismatch	0.92 (0.47–1.81)	0.80			1.25 (0.70–2.24)	0.45	1.59 (0.79–3.18)	0.19	0.68 (0.28–1.64)	0.39	0.45 (0.09–2.10)	0.31
Minor mismatch	1.23 (0.69–2.20)	0.49			1.73 (1.03–2.90)	**0.04**	2.22 (1.18–4.17)	**0.014**	0.52 (0.23–1.21)	0.13	0.66 (0.24–1.81)	0.42
Bidirectional mismatch	1.25 (0.65–2.42)	0.50			0.83 (0.40–1.70)	0.61	1.22 (0.55–2.69)	0.62	1.87 (0.98–3.59)	0.06	0.94 (0.29–3.10)	0.92
CMV antibody—no. (%)												
Both negative	1.07 (0.48–2.38)		1		1				1			
Either positive	2.84 (1.14–7.06)	**0.03**	2.33 (0.67–8.01)	0.18	1.39 (0.62–3.14)	0.43			2.62 (0.78–8.83)	0.12		
HCT-CI score—no. (%)												
0	1				1				1			
≥1	1.45 (0.91–2.31)	0.11			1.11 (0.68–1.81)	0.69			1.55 (0.85–2.83)	0.15		
ECOG PS—no. (%)												
0	1				1				1			
≥1	1.21 (0.76–1.92)	0.42			1.05 (0.64–1.73)	0.84			0.98 (0.53–1.80)	0.94		
Conditioning regimen intensity—no. (%)												
MAC	1		1		1				1		1	
RIC	1.56 (0.98–2.48)	0.06	0.89 (0.42–1.86)	0.75	0.74 (0.43–1.26)	0.27			2.22 (1.21–4.07)	**0.01**	0.92 (0.37–2.27)	0.85
GVHD prophylaxis—no. (%)												
CNI + MTX	1				1				1			
Others	1.33 (0.66–2.68)	0.42			1.06 (0.47–2.41)	0.88			1.28 (0.48–3.36)	0.62		
Engraftment day—no. (%)												
<day 18	1				1				1			
≥day 18	1.75 (0.88–3.49)	0.11			1.16 (0.64–2.09)	0.63			2.19 (0.76–6.25)	0.15		
Grade 2–4 acute GVHD—no. (%)												
No	1				1				1		1	
Yes	1.34 (0.79–2.29)	0.28			0.70 (0.41–1.19)	0.18			2.66 (1.16–6.13)	**0.02**	2.86 (1.16–7.08)	**0.02**
TMA—no. (%)												
No	1				1				1			
Yes	1.30 (0.41–4.17)	0.66			0.38 (0.05–2.75)	0.33			1.94 (0.50–7.56)	0.34		
SOS—no. (%)												
No	1				1				1		1	
Yes	1.73 (0.42–7.13)	0.45			1.37 (0.35–5.44)	0.65			0.00 (0.00–0.00)	**<0.001**	0.00 (0.00–0.00)	**<0.001**
CMV viremia—no. (%)												
No	1				1				1		1	
Yes	1.45 (0.85–2.48)	0.17			0.69 (0.41–1.16)	0.16			2.53 (1.13–5.67)	**0.02**	1.98 (0.79–5.00)	0.15
Fluid retention—no. (%) Locations												
No PL/PT/PC effusion	1				1		1		1			
PL effusion	2.96 (1.73–5.05)	**<0.001**			1.42 (0.84–2.38)	0.19			2.32 (1.09–4.97)	**0.03**		
PT effusion	2.29 (1.34–3.91)	**0.002**			1.18 (0.70–2.00)	0.54			2.44 (1.14–5.23)	**0.02**		
PC effusion	4.63 (2.61–8.23)	**<0.001**			1.79 (0.98–3.26)	**0.06**	1.57 (0.91–2.71)	0.10	2.33 (0.97–5.61)	0.06		
Number of affected cavities												
None	1		1		1				1		1	
Single cavity	2.60 (1.24–5.44)	**0.01**	2.96 (1.30–6.76)	**0.01**	1.29 (0.68–2.46)	0.44			1.13 (0.42–2.99)	0.81	2.36 (0.86–6.47)	0.10
Double cavities	3.12 (1.56–6.25)	**0.001**	2.77 (1.30–5.90)	**0.008**	1.25 (0.69–2.27)	0.46			1.60 (0.69–3.73)	0.27	1.96 (0.73–5.29)	0.18
Triple cavities	7.94 (3.59–17.56)	**<0.001**	6.69 (2.77–16.15)	**<0.001**	1.42 (0.63–3.19)	0.4			3.13 (1.13–8.65)	**0.03**	6.27 (1.40–28.13)	**0.02**

Bold values indicate values with statistical significance

*AML* acute myeloid leukemia, *BMT* bone marrow transplantation, *CB* cord blood, *CBT* cord blood transplantation, *CMV* cytomegalovirus, *CNI* calcineurin inhibitor, *DRI* disease risk index, *ECOG PS* Eastern Cooperative Oncology Group Performance Status Scale, *GVHD* graft-versus-host disease, *HCT-CI* Hematopoietic Cell Transplantation-Comorbidity Index, *MAC* myeloablative conditioning, *MMRD* HLA-mismatched related donor, *MMUD* HLA-mismatched unrelated donor, *MRD* HLA-matched related donor, *MTX* methotrexate, *MUD* HLA-matched unrelated donor, *PBSCT* peripheral blood stem cell transplantation, *RIC* reduced intensity conditioning, *SOS* sinusoidal obstruction syndrome, *TMA* thrombotic microangiopathy

The impact of the number of effusion-positive cavities on OS showed additive effect; effusions in single, double, and triple cavities were associated with OS with the HR of 2.96 (95% CI, 1.30–6.67; *P* = 0.01), 2.77 (95% CI, 1.30–5.90; *P* = 0.008), and 6.69 (95% CI, 2.77–16.1; *P* < 0.001), respectively, on multivariate analysis (overall *P* < 0.001; Table 2). The 2-year OS rate of patients with effusions at no, single, double, and triple cavities were 86.1% (95% CI, 76.3–92.1), 60.0% (95% CI, 38.3–76.2), 59.6% (95% CI, 39.0–75.3), and 18.8% (95% CI, 3.1–44.7), respectively (overall *P* < 0.001; Fig. 2d).

To investigate the relationship between cavity effusions and OS, regression analysis of CIR and NRM was performed, and the primary causes of death in patients with or without cavity effusions were examined. Neither PL, PT, nor PC effusions were associated with CIR (Table 2). Concomitantly, the number of effusion-positive cavities was not associated with CIR (overall *P* = 0.43; Fig. 2e). In contrast, PL and PT effusions were significantly associated with NRM on univariate analysis. Although a number of effusion-positive cavities were not significantly associated with NRM overall (*P* = 0.08), effusion in triple cavities was significantly associated with NRM on multivariate analysis (HR, 6.27; 95% CI, 1.40–28.1; *P* = 0.02; Fig. 2f; Table 2). Regarding the primary causes of death, patients with multiple effusion-positive cavities were more likely to die due to infection than those with none or single effusion-positive cavities (16 out of 41 deaths vs. 3 out of 32 deaths: Table 3).

**Table 3:** Primary death causes stratified by the number of effusion-positive cavities within day 100 following allo-HSCT

	None or single	Multiple	Total
Disease recurrence—no. (%)	10 (32.3)	9 (22.0)	19 (26.4)
Infection—no. (%)	3 (9.7)	16 (39.0)	19 (26.4)
Pulmonary complications other than infection—no. (%)	4 (12.9)	4 (9.8)	8 (11.1)
Organ failure—no. (%)	3 (9.7)	2 (4.9)	5 (6.9)
Bleeding—no. (%)	3 (9.7)	2 (4.9)	5 (6.9)
Acute GVHD—no. (%)	2 (6.5)	3 (7.3)	5 (6.9)
TMA—no. (%)	2 (6.5)	1 (2.4)	3 (4.2)
SOS—no. (%)	1 (3.2)	1 (2.4)	2 (2.8)
Chronic GVHD—no. (%)	0 (0.0)	2 (4.9)	2 (2.8)
Aging—no. (%)	1 (3.2)	0 (0.0)	1 (1.4)
Trauma—no. (%)	1 (3.2)	0 (0.0)	1 (1.4)
Graft failure—no. (%)	0 (0.0)	1 (2.4)	1 (1.4)
Unknown—no. (%)	1 (3.2)	0 (0.0)	1 (1.4)
Total—no. (%)	31 (100.0)	41 (100.0)	72 (100.0)

*GVHD* graft-versus-host disease, *SOS* sinusoidal obstruction syndrome, *TMA* thrombotic microangiopathy

Taken together, cavity effusions following allo-HSCT were strongly and independently associated with inferior survival in a manner dependent on the number of effusion-positive cavities.

### Characteristics and conditions associated with cavity effusions

Knowing the association between effusion and poor prognosis, we set out to characterize the cavity effusions in terms of its size and associated allo-HSCT conditions. Out of 106 PL effusions which were found for the first time in each patient after allo-HSCT, 76 (71.7%) were bilateral. Left-sided effusions (31, 29.2%) were slightly more common than right-sided effusions (22, 20.8%). The majority of the PL effusions was small (81 (76.4%) cases), even when restricted to the acute phase in the first 100 days following allo-HSCT (57 (79.2%) out of 72 cases of PL effusion during the first 100 days following allo-HSCT), while the minority was moderate sized (14 (13.2%) cases during the whole observation period, and 6 (8.3%) cases during the first 100 days following allo-HSCT; others lack original imaging data). Similarly, small PT effusions were more frequently encountered than moderate PT effusions during the whole observation period and during the first 100 days (67 vs. 21, and 57 vs. 15, respectively). During the first 100 days, 6 cases of moderate PL effusions and 15 cases of PT effusions were only found in patients with multiple sites of effusions. The albumin level was 2.8 g/dL (range, 1.4–5.0 g/dL), 2.8 g/dL (range, 1.4–4.8 g/dL), and 2.8 g/dL (range, 1.8–4.4 g/dL) when PL, PT, and PC effusions were first observed, respectively (reference range, 4.1–5.1 g/dL). These values were significantly lower compared to albumin level on day 0 (median, 3.6 mg/dL for all 178 patients; *P* < 0.001 for each effusion). By D100, the proportion of patients who were administered systemic steroids was higher in patients with effusions(s) than those without: 22 out of 92 (23.9%) patients vs. 9 out of 86 (10.5%) patients (*P* = 0.03).

Primarily associated conditions at the time of effusions were next investigated. Cavity effusions were associated with varying conditions depending on the time of onset and whether they were recurrent or not (Table 4). While volume overload/heart failure, SOS, and engraftment syndrome were predominantly associated with effusions within the first 100 days, pneumonia was associated with PL effusions both in the acute (14 cases) and chronic (8 cases) phases after allo-HSCT. Of note, no specific causes of effusions were identified in a substantial proportion of patients. To reveal possible associations with these complications and effusions, the number of patients diagnosed with specific complications by D100 were compared with or without effusion in at least one cavity by D100 (Supplementary Table 1). The proportion of patients who experienced engraftment syndrome, thrombotic microangiopathy, and bacterial infection was significantly higher in those with CT-identified effusion(s).

**Table 4:** Conditions associated with cavity effusions

	First time			Recurrent > 100 days
	≤100 days	>100 days	Total	
PL effusion				
Pneumonia^a^—no. (%)	14 (19.4)	8 (23.5)	22 (20.8)	18 (47.4)
Volume overload/heart failure—no. (%)	11 (15.3)	4 (11.8)	15 (14.2)	3 (7.9)
NIPC—no. (%)	2 (2.8)	8 (23.5)	10 (9.4)	3 (7.9)
Kidney injury excluding TMA—no. (%)	2 (2.8)	1 (2.9)	3 (2.8)	1 (2.6)
Sepsis—no. (%)	0 (0.0)	0 (0.0)	0 (0.0)	1 (2.6)
Engraftment syndrome—no. (%)	2 (2.8)	0 (0.0)	2 (1.9)	0 (0.0)
SOS—no. (%)	1 (1.4)	0 (0.0)	1 (0.9)	0 (0.0)
Malignancy—no. (%)	0 (0.0)	1 (2.9)	1 (0.9)	3 (7.9)
TMA—no. (%)	2 (2.8)	0 (0.0)	2 (1.9)	1 (2.6)
GI GVHD—no. (%)	0 (0.0)	0 (0.0)	0 (0.0)	1 (2.6)
Others—no. (%)	0 (0.0)	0 (0.0)	0 (0.0)	2 (5.3)
Not determined—no. (%)	38 (52.8)	12 (35.3)	50 (47.2)	5 (13.6)
Total—no. (%)	72 (100.0)	34 (100.0)	106 (100.0)	38 (100.0)
PT effusion				
Volume overload/heart failure—no. (%)	9 (12.5)	1 (6.3)	10 (11.4)	2 (8.3)
Engraftment syndrome—no. (%)	6 (8.3)	0 (0.0)	6 (6.8)	0 (0.0)
SOS—no. (%)	3 (4.2)	0 (0.0)	3 (3.4)	0 (0.0)
TMA—no. (%)	2 (2.8)	0 (0.0)	2 (2.3)	1 (4.2)
Kidney injury excluding TMA—no. (%)	1 (1.4)	1 (6.3)	2 (2.3)	1 (4.2)
GI GVHD—no. (%)	2 (2.8)	0 (0.0)	2 (2.3)	2 (8.3)
Ileus, enteritis—no. (%)	5 (6.9)	1 (6.3)	6 (6.8)	1 (4.2)
Sepsis—no. (%)	0 (0.0)	0 (0.0)	0 (0.0)	1 (4.2)
Others—no. (%)	0 (0.0)	0 (0.0)	0 (0.0)	2 (8.3)
Not determined—no. (%)	44 (61.1)	13 (81.3)	57 (64.8)	14 (58.3)
Total—no. (%)	72 (100.0)	16 (100.0)	88 (100.0)	24 (100.0)
PC effusion				
Volume overload/heart failure—no. (%)	6 (16.2)	0 (0.0)	6 (11.3)	1 (9.1)
TMA—no. (%)	2 (5.4)	0 (0.0)	2 (3.8)	1 (9.1)
Kidney injury excluding TMA—no. (%)	0 (0.0)	1 (6.3)	1 (1.9)	0 (0.0)
Sepsis—no. (%)	0 (0.0)	0 (0.0)	0 (0.0)	1 (9.1)
GI GVHD—no. (%)	0 (0.0)	0 (0.0)	0 (0.0)	1 (9.1)
Not determined—no. (%)	29 (78.4)	15 (93.8)	53 (83.2)	7 (63.6)
Total—no. (%)	37 (100.0)	16 (100.0)	53 (100.0)	11 (100.0)

*GVHD* graft-versus-host disease, *NIPC* non-infectious pulmonary complications, *SOS* sinusoidal obstruction syndrome, *TMA* thrombotic microangiopathy

^a^Cases with determined causative microbes of pneumonia were as follows: multi-drug-resistant *Pseudomonas aeruginosa* was identified from sputum culture in two cases, *Mycobacterium tuberculosis* DNA from sputum in one case, *Stenotrophomonas maltophilia* from sputum culture in one case, and CMV DNA from sputum in one case

As for 42 patients with recurrent effusions beyond D100, 37 were hospitalized for underlying conditions. Eighteen patients were receiving immunosuppressants for active chronic GVHD. A total of 18 cases with PL effusion were associated with pneumonia, although the causal microbes were not detected in all cases. Volume overload or heart failure was scarcely represented in this period.

### Predictive factors for cavity effusions

Finally, to identify predictive factors for cavity effusions, various transplant-related variables were subjected to univariate and multivariate analysis. During the whole observation period, high/very high refined DRI was associated with effusions on univariate analysis, regardless of their location (Supplementary Table 2). Except for PC effusion, high/very high refined DRI was associated with effusions also on multivariate analysis.

Predictive factors for effusions depending on the number of effusion-positive cavities during the acute phase (≤D100) following allo-HSCT were next investigated. Higher refined DRI was consistently associated both with single and with multiple cavities of effusions on multivariate analysis (Table 5). These patients with higher DRI were significantly associated with older age at transplantation, higher HCT-CI scores, and higher pre-transplant EASIX (Supplementary Table 3). Although not statistically significant, worse ECOG PS and shorter time from diagnosis to transplantation despite comparable cycles of cytotoxic chemotherapy were also observed in patients with higher DRI.

**Table 5:** Transplantation variables associated with cavity effusions according to the number of effusion-positive cavities

Characteristics	Single cavity				Double cavities				Triple cavities			
	Univariate analysis		Multivariate analysis		Univariate analysis		Multivariate analysis		Univariate analysis		Multivariate analysis	
	HR (95% CI)	*P* values	HR (95% CI)	*P* values	HR (95% CI)	*P* values	HR (95% CI)	*P* values	HR (95% CI)	*P* values	HR (95% CI)	*P* values
Age—no. (%)												
<50	1				1		1		1			
≥50	1.09 (0.72–1.63)	0.69			1.64 (1.00–2.69)	0.05	1.26 (0.59–2.70)	0.56	1.70 (0.76–3.76)	0.19		
Recipient sex—no. (%)												
Male	1				1				1			
Female	0.95 (0.63–1.45)	0.81			0.68 (0.41–1.14)	0.15			0.76 (0.33–1.75)	0.52		
Sex match between recipient and donor—no. (%)												
Match	1				1				1			
Male to female	0.80 (0.45–1.41)	0.44			0.92 (0.48–1.76)	0.79			0.96 (0.33–2.79)	0.95		
Female to male	1.01 (0.61–1.67)	0.98			1.41 (0.80–2.49)	0.24			1.35 (0.57–3.23)	0.50		
Diagnosis—no. (%)												
AML	1				1				1		1	
Other myeloid neoplasms	1.43 (0.88–2.32)	0.15			1.24 (0.69–2.24)	0.47			1.41 (0.56–3.56)	0.47	1.33 (0.43–4.08)	0.62
Lymphoid and ambiguous lineage neoplasms	1.35 (0.88–2.06)	0.17			1.40 (0.85–2.32)	0.19			0.81 (0.34–1.92)	0.63	0.87 (0.33–2.28)	0.78
Inborn errors of metabolism	0.47 (0.16–1.34)	0.16			0.33 (0.08–1.37)	0.13			0.00 (0.00–0.00)	**<0.001**	0.00 (0.00–0.00)	**<0.001**
Others	2.99 (0.59–15.05)	0.19			2.00 (0.36–11.27)	0.43			2.18 (0.24–19.74)	0.49	4.70 (0.24–90.46)	0.31
Refined DRI—no. (%)												
Low/intermediate	1		1		1		1		1		1	
High/very high	1.71 (1.14–2.57)	**0.01**	1.71 (1.14–2.57)	**0.01**	2.13 (1.30–3.48)	**0.003**	1.81 (1.04–3.17)	**0.04**	2.79 (1.24–6.30)	**0.01**	2.62 (1.10–6.25)	**0.03**
Donor type—no. (%)												
MRD/MUD	1				1				1			
MMRD/MMUD	1.35 (0.86–2.13)	0.19			1.06 (0.60–1.88)	0.84			0.97 (0.39–2.43)	0.95		
CB	1.08 (0.65–1.79)	0.76			1.13 (0.62–2.05)	0.69			1.07 (0.41–2.79)	0.88		
Cell source—no. (%)												
PBSC	1				1				1			
BM	1.06 (0.69–1.63)	0.79			1.24 (0.74–2.09)	0.42			0.96 (0.43–2.16)	0.93		
CB	1.08 (0.65–1.79)	0.76			1.13 (0.62–2.05)	0.69			1.07 (0.41–2.79)	0.88		
ABO—no. (%)												
Match	1				1				1		1	
Major mismatch	1.14 (0.68–1.91)	0.62			1.23 (0.67–2.24)	0.50			2.11 (0.91–4.90)	0.08	2.29 (0.83–6.35)	0.11
Minor mismatch	0.76 (0.46–1.27)	0.30			1.21 (0.69–2.12)	0.51			0.86 (0.32–2.28)	0.76	0.93 (0.25–3.51)	0.91
Bidirectional mismatch	1.04 (0.59–1.81)	0.90			0.94 (0.48–1.83)	0.85			0.66 (0.21–2.14)	0.49	0.69 (0.20–2.45)	0.57
CMV antibody—no. (%)												
Both negative	1				1		1		1			
Either positive	1.67 (0.81–3.47)	0.17			2.29 (0.91–5.76)	0.08	2.07 (0.76–5.64)	0.15	2.06 (0.48–8.73)	0.33		
HCT-CI score—no. (%)												
0	1				1				1		1	
≥1	1.25 (0.83–1.88)	0.28			1.37 (0.84–2.22)	0.21			2.16 (0.97–4.79)	0.06	1.75 (0.73–4.19)	0.21
ECOG PS—no. (%)												
0	1				1		1		1			
≥1	1.35 (0.90–2.03)	0.14			1.90 (1.17–3.09)	**0.01**	1.76 (0.99–3.15)	0.06	1.82 (0.83–3.99)	0.14		
Conditioning regimen intensity—no. (%)												
MAC	1				1		1		1			
RIC	1.38 (0.92–2.07)	0.12			1.66 (1.02–2.70)	**0.04**	1.14 (0.57–2.27)	0.71	1.51 (0.69–3.29)	0.30		
GVHD prophylaxis—no. (%)												
CNI + MTX	1				1				1			
Others	1.01 (0.56–1.84)	0.96			0.83 (0.38–1.81)	0.64			0.75 (0.18–3.11)	0.69		

Bold values indicate values with statistical significance

*AML* acute myeloid leukemia, *BMT* bone marrow transplantation, *CB* cord blood, *CBT* cord blood transplantation, *CNI* calcineurin inhibitor, *DRI* disease risk index, *ECOG PS* Eastern Cooperative Oncology Group Performance Status Scale, *HCT-CI* Hematopoietic Cell Transplantation-Comorbidity Index, *MAC* myeloablative conditioning, *MMRD* HLA-mismatched related donor, *MMUD* HLA-mismatched unrelated donor, *MRD* HLA-matched related donor, *MTX* methotrexate, *MUD* HLA-matched unrelated donor, *PBSCT* peripheral blood stem cell transplantation, *RIC* reduced intensity conditioning

Together, effusions observed following allo-HSCT are predominantly small, and their specific causes could not be determined in most cases. However, higher disease risk posed a risk to the development of effusions following allo-HSCT, suggesting effusions serve as proxies for severe primary disease status.

## Discussion

In this study, we comprehensively analyzed CT-detected effusions in body cavities following allo-HSCT. We revealed that cavity effusions in the acute phase post-allo-HSCT are associated with adverse outcome even when most of them were small or with undermined etiology. Our findings build on the existing evidence of post-transplant fluid retention to propose that the number of effusion-positive cavities serves as proxy for survival.

In our retrospective analysis, most effusions occurred in the acute phase following allo-HSCT, with the median cumulative incidence of PL, PT, and PC effusions reaching 41.0%, 40.4%, and 20.8% at D100, respectively. The cumulative incidence of PL effusion was much higher than in the previous study, reporting 9.9% (95% CI, 7.7–12.5%) at 1 year [4]. The discrepancy would be explained by the definition of PL effusion; in that study, PL effusions were included in the analysis only when symptomatic, while in our study, all PL effusions detected on CT were included. Assessing whether PL effusion is symptomatic or not could be arbitrary, especially in a retrospective study. Therefore, we adopted CT-based judgment on the existence of effusions, in line with several other studies [5, 6, 8, 25], showing the incidence of PT and PC effusions 27% [6] and 20–26% [5, 8] in adult patients.

The unique aspect of our study was that effusions in three cavities were examined in the same cohort simultaneously, enabling the comparative and combinatory assessment of the incidence and outcome of effusions in each cavity. First, the incidence of PL and PT effusions was higher than PC effusion. Second, higher disease risk was associated with the development of PL and PT, but not of PC, in multivariate analysis. Third, although not mutually exclusive and thus denied direct comparison, PC effusions tended to be associated with a worse prognosis than PL or PT effusions, which showed similar prognostic impact. These findings suggest that PL and PT cavities might be more sensitive to fluid and disease burden than PC cavities. This might reflect the distinct pathophysiological basis among different body cavities or simply that PL and PT effusions could be more easily detected on CT than PC effusion. Another interesting observation was that the number of effusion-positive cavities showed an additive adverse effect on the transplantation outcome. This finding is an important confirmation that fluid retention is associated with worse survival. Inferior survival in these effusion-positive patients was attributed to increased NRM, particularly due to infection. This might partly be a consequence of higher proportion of patients who were administered steroids. Rondón et al. reported that fluid overload, defined by weight gain immediately following allo-HSCT, was associated with higher NRM and worse OS [26]. However, the immunological disturbance post-allo-HSCT adds complexity to a mere fluid overload in traditional intensive care unit settings [27]. In the allo-HSCT setting, the complex combinations of fluid overload, endothelial damage, and inflammatory conditions may all contribute to cavity effusions in a single patient. Indeed, the proportion of patients with engraftment syndrome, thrombotic microangiopathy, and bacterial infection was significantly higher in those with effusion(s) in our analysis. Our findings extend the current knowledge to propose that cavity effusion serves as a qualitative surrogate for these multifactorial conditions and as a quantitative proxy for survival.

Further, we showed that patients with higher disease risk were associated with increased risk for post-HSCT effusions. This is a stark contrast to patients with inborn errors of metabolisms undergoing allo-HSCT, who were at minimal risk for effusions. In patients with malignancy, increased vascular permeability and inflammatory status could contribute to the emergence of effusions [28]. Indeed, in our series, pre-transplant EASIX, a recently defined indicator of endothelial injury [22], was significantly higher in patients with higher disease risk. This might be attributable to older age at transplantation, more comorbidities, and history of intensive chemotherapies in a relatively shorter time from diagnosis, all of which were also observed in this subset of patients. Our results therefore indicate the emergence of post-transplant cavity effusions significantly relies on each patient’s background before allo-HSCT.

We acknowledge that our study harbors limitations inherent to a retrospective study. CT was conducted only upon some clinical suspicion of the patient’s status. Therefore, the true incidence and timing of effusions might have been underestimated. Indeed, CT examinations were more often performed in patients with effusion than in those without effusion in shorter period of observation period (9 CT scans in 430 days of observation period and 5 CT scans in 1413 days, respectively; all represented in median). CT scans were also conducted more frequently in patients with effusion by D100, compared to those without (median 4 and 2, respectively). Although it is possible that patients with cavity effusions were more closely monitored, but it is an equally reasonable postulation that cavity effusions were detected because of the intense monitoring. Precise documentation of the temporal emergence of cavity effusions would also guide the fluid and immunosuppressant management post-allo-HSCT. Furthermore, effusions arise from a variety of causes, each requiring different management. Effusion with undermined causes in our cohort therefore presents an important knowledge gap. A prospective study design with standardized time points of CT and inclusion of body weight and laboratory data would be necessary to further characterize the fluid dynamics following allo-HSCT.

In conclusion, we showed that fluid retention observed as effusions in body cavities is a frequent complication associated with inferior survival after allo-HSCT. The number of effusion-positive cavities showed an additive adverse effect on the transplantation outcome. We expect further studies to determine the fluid dynamics in post-transplantation setting.

## Supplementary Information

Below is the link to the electronic supplementary material.

Supplementary file1 (DOCX 17 KB)

Supplementary file2 (DOCX 33 KB)

Supplementary file3 (DOCX 18 KB)

## Data Availability

Relevant data are available upon reasonable request to the corresponding author.
