# Supplementary material for: Prognostic impact of effusion in multiple body cavities after allogeneic hematopoietic stem cell transplantation
Source: Int J Hematol. 2025 Mar 3;121(6):833–47. doi: 10.1007/s12185-025-03949-7 (PMC12106147; doi:10.1007/s12185-025-03949-7)
Supplement: Supplementary file 1 — Supplementary file1 (DOCX 17 KB) [file 12185_2025_3949_MOESM1_ESM.docx]

| **Supplementary Table 1. Number of patients with specific complications stratified by the presence or absence of effusion(s) by day 100.** | | | | |
| --- | --- | --- | --- | --- |
| Complications | | Effusion by day 100 | |  |
|  |  | Negative (*n*=86) | Positive (*n*=92) | *P* values |
| PIR - no. (%) | No | 86 (100.0) | 92 (100.0) |  |
| ES - no. (%) | No | 86 (100.0) | 84 (91.3) | **0.007** |
|  | Yes | 0 (0.0) | 8 (8.7) |  |
| Grade 2-4 acute GVHD - no. (%) | No | 51 (59.3) | 50 (54.3) | 0.51 |
|  | Yes | 35 (40.7) | 42 (45.7) |  |
| TMA - no. (%) | No | 84 (97.7) | 80 (87.0) | **0.008** |
|  | Yes | 2 (2.3) | 12 (13.0) |  |
| SOS - no. (%) | No | 85 (98.8) | 85 (92.4) | 0.07 |
|  | Yes | 1 (1.2) | 7 (7.6) |  |
| Bacterial infection - no. (%) | No | 35 (40.7) | 24 (26.1) | **0.04** |
|  | Yes | 51 (59.3) | 68 (73.9) |  |
| CMV infection - no. (%) | No | 83 (96.5) | 82 (89.1) | 0.06 |
|  | Yes | 3 (3.5) | 10 (10.9) |  |
| CMV, cytomegalovirus; ES, engraftment syndrome; GVHD, graft-versus-host disease; PIR, pre-engraftment immune reaction; SOS, sinusoidal obstruction syndrome; and TMA, thrombotic microangiopathy. | | | | |
