# Supplementary material for: Prognostic impact of effusion in multiple body cavities after allogeneic hematopoietic stem cell transplantation
Source: Int J Hematol. 2025 Mar 3;121(6):833–47. doi: 10.1007/s12185-025-03949-7 (PMC12106147; doi:10.1007/s12185-025-03949-7)
Supplement: Supplementary file 2 — Supplementary file2 (DOCX 33 KB) [file 12185_2025_3949_MOESM2_ESM.docx]

| **Supplementary Table 2. Transplantation variables associated with cavity effusions.** | | | | | | | | | | | | | | | | | | |
| --- | --- | --- | --- | --- | --- | --- | --- | --- | --- | --- | --- | --- | --- | --- | --- | --- | --- | --- |
| Characteristics | | PL effusion | | | | |  | PT effusion | | | | |  | PC effusion | | | | |
|  |  | Univariate analysis | |  | Multivariate analysis | |  | Univariate analysis | |  | Multivariate analysis | |  | Univariate analysis | |  | Multivariate analysis | |
|  |  | HR (95% CI) | *P* values |  | HR (95% CI) | *P* values |  | HR (95% CI) | *P* values |  | HR (95% CI) | *P* values |  | HR (95% CI) | *P* values |  | HR (95% CI) | *P* values |
| Age - no. (%) | |  |  |  |  |  |  |  |  |  |  |  |  |  |  |  |  |  |
|  | < 50 | 1 |  |  | 1 |  |  | 1 |  |  |  |  |  | 1 |  |  | 1 |  |
|  | ≥ 50 | 1.52 (1.04-2.22) | **0.03** |  | 0.97 (0.52-1.79) | 0.91 |  | 1.07 (0.71-1.62) | 0.75 |  |  |  |  | 1.87 (1.08-3.23) | **0.03** |  | 1.54 (0.67-3.57) | 0.31 |
| Recipient sex - no. (%) | |  |  |  |  |  |  |  |  |  |  |  |  |  |  |  |  |  |
|  | Male | 1 |  |  |  |  |  | 1 |  |  |  |  |  | 1 |  |  |  |  |
|  | Female | 0.77 (0.51-1.15) | 0.20 |  |  |  |  | 0.87 (0.57-1.33) | 0.52 |  |  |  |  | 1.00 (0.58-1.73) | 1.00 |  |  |  |
| Sex match between recipient and donor - no. (%) | |  |  |  |  |  |  |  |  |  |  |  |  |  |  |  |  |  |
|  | Match | 1 |  |  |  |  |  | 1 |  |  |  |  |  | 1 |  |  |  |  |
|  | Male to female | 0.73 (0.41-1.30) | 0.29 |  |  |  |  | 0.96 (0.55-1.66) | 0.87 |  |  |  |  | 0.51 (0.20-1.28) | 0.15 |  |  |  |
|  | Female to male | 1.28 (0.83-1.97) | 0.26 |  |  |  |  | 1.15 (0.70-1.88) | 0.59 |  |  |  |  | 1.08 (0.56-2.06) | 0.82 |  |  |  |
| Diagnosis - no. (%) | |  |  |  |  |  |  |  |  |  |  |  |  |  |  |  |  |  |
|  | AML | 1 |  |  | 1 |  |  | 1 |  |  | 1 |  |  | 1 |  |  | 1 |  |
|  | Other myeloid neoplasms | 1.64 (1.09-2.45) | **0.02** |  | 1.53 (0.90-2.60) | 0.12 |  | 1.59 (1.00-2.54) | 0.05 |  | 1.67 (0.94-2.95) | 0.08 |  | 1.49 (0.80-2.77) | 0.20 |  | 1.26 (0.61-2.57) | 0.53 |
|  | Lymphoid and ambiguous lineage neoplasms | 1.26 (0.83-1.91) | 0.27 |  | 1.69 (1.01-2.83) | 0.05 |  | 1.13 (0.73-1.77) | 0.58 |  | 1.57 (0.96-2.57) | 0.08 |  | 0.60 (0.31-1.14) | 0.12 |  | 0.70 (0.33-1.48) | 0.35 |
|  | Inborn errors of metabolism | 0.23 (0.07-0.73) | **0.01** |  | 0.44 (0.11-1.71) | 0.23 |  | 0.65 (0.26-1.65) | 0.36 |  | 1.17 (0.43-3.21) | 0.76 |  | 0.18 (0.03-1.26) | 0.08 |  | 0.18 (0.03-1.23) | 0.08 |
|  | Others | 4.49 (1.80-11.22) | **0.001** |  | 6.04 (2.57-14.19) | **< 0.001** |  | 2.12 (0.63-7.11) | 0.22 |  | 4.20 (0.71-24.92) | 0.11 |  | 1.99 (0.46-8.56) | 0.36 |  | 3.45 (0.81-14.75) | 0.10 |
| Refined DRI - no. (%) | |  |  |  |  |  |  |  |  |  |  |  |  |  |  |  |  |  |
|  | Low/intermediate | 1 |  |  | 1 |  |  | 1 |  |  | 1 |  |  | 1 |  |  | 1 |  |
|  | High/very high | 2.36 (1.61-3.45) | **< 0.001** |  | 2.20 (1.41-3.42) | **< 0.001** |  | 2.11 (1.39-3.21) | **< 0.001** |  | 2.12 (1.37-3.27) | **< 0.001** |  | 1.76 (1.03-3.00) | **0.04** |  | 1.28 (0.69-2.35) | 0.43 |
| Donor type - no. (%) | |  |  |  |  |  |  |  |  |  |  |  |  |  |  |  |  |  |
|  | MRD/MUD | 1 |  |  |  |  |  | 1 |  |  |  |  |  | 1 |  |  |  |  |
|  | MMRD/MMUD | 0.89 (0.57-1.40) | 0.62 |  |  |  |  | 1.07 (0.66-1.74) | 0.79 |  |  |  |  | 1.25 (0.69-2.26) | 0.47 |  |  |  |
|  | CB | 1.06 (0.66-1.71) | 0.81 |  |  |  |  | 1.04 (0.62-1.73) | 0.89 |  |  |  |  | 1.06 (0.54-2.08) | 0.86 |  |  |  |
| Cell source - no. (%) | |  |  |  |  |  |  |  |  |  |  |  |  |  |  |  |  |  |
|  | PBSC | 1 |  |  |  |  |  | 1 |  |  |  |  |  | 1 |  |  |  |  |
|  | BM | 1.09 (0.74-1.62) | 0.66 |  |  |  |  | 1.13 (0.73-1.75) | 0.59 |  |  |  |  | 1.05 (0.60-1.84) | 0.85 |  |  |  |
|  | CB | 1.06 (0.66-1.71) | 0.81 |  |  |  |  | 1.04 (0.62-1.73) | 0.89 |  |  |  |  | 1.06 (0.54-2.08) | 0.86 |  |  |  |
| ABO - no. (%) | |  |  |  |  |  |  |  |  |  |  |  |  |  |  |  |  |  |
|  | Match | 1 |  |  |  |  |  | 1 |  |  |  |  |  | 1 |  |  |  |  |
|  | Major mismatch | 1.14 (0.71-1.84) | 0.59 |  |  |  |  | 1.11 (0.65-1.87) | 0.71 |  |  |  |  | 1.42 (0.76-2.67) | 0.27 |  |  |  |
|  | Minor mismatch | 1.11 (0.73-1.71) | 0.62 |  |  |  |  | 0.90 (0.55-1.46) | 0.66 |  |  |  |  | 0.91 (0.46-1.77) | 0.77 |  |  |  |
|  | Bidirectional mismatch | 1.07 (0.65-1.76) | 0.80 |  |  |  |  | 1.00 (0.56-1.80) | 1.00 |  |  |  |  | 0.76 (0.35-1.66) | 0.49 |  |  |  |
| CMV antibody - no. (%) | |  |  |  |  |  |  |  |  |  |  |  |  |  |  |  |  |  |
|  | Both negative | 1 |  |  | 1 |  |  | 1 |  |  |  |  |  | 1 |  |  | 1 |  |
|  | Either positive | 1.99 (1.04-3.82) | **0.04** |  | 1.31 (0.64-2.68) | 0.47 |  | 1.63 (0.81-3.30) | 0.17 |  |  |  |  | 2.34 (0.86-6.38) | 0.10 |  | 1.54 (0.57-4.14) | 0.39 |
| HCT-CI score - no. (%) | |  |  |  |  |  |  |  |  |  |  |  |  |  |  |  |  |  |
|  | 0 | 1 |  |  | 1 |  |  | 1 |  |  |  |  |  | 1 |  |  |  |  |
|  | ≥ 1 | 1.43 (0.98-2.08) | 0.07 |  | 1.20 (0.78-1.83) | 0.41 |  | 1.30 (0.86-1.97) | 0.21 |  |  |  |  | 1.38 (0.81-2.36) | 0.24 |  |  |  |
| ECOG PS - no. (%) | |  |  |  |  |  |  |  |  |  |  |  |  |  |  |  |  |  |
|  | 0 | 1 |  |  |  |  |  | 1 |  |  |  |  |  | 1 |  |  | 1 |  |
|  | ≥ 1 | 1.27 (0.87-1.86) | 0.22 |  |  |  |  | 1.37 (0.90-2.07) | 0.14 |  |  |  |  | 1.59 (0.93-2.71) | 0.09 |  | 1.95 (1.10-3.45) | **0.02** |
| Conditioning regimen intensity - no. (%) | |  |  |  |  |  |  |  |  |  |  |  |  |  |  |  |  |  |
|  | MAC | 1 |  |  | 1 |  |  | 1 |  |  | 1 |  |  | 1 |  |  | 1 |  |
|  | RIC | 1.96 (1.35-2.86) | **< 0.001** |  | 2.02 (1.16-3.52) | **0.01** |  | 1.42 (0.94-2.15) | 0.10 |  | 1.30 (0.83-2.05) | 0.25 |  | 1.66 (0.97-2.83) | 0.06 |  | 1.13 (0.52-2.43) | 0.76 |
| GVHD prophylaxis - no. (%) | |  |  |  |  |  |  |  |  |  |  |  |  |  |  |  |  |  |
|  | CNI+MTX | 1 |  |  |  |  |  | 1 |  |  |  |  |  | 1 |  |  |  |  |
|  | Others | 1.38 (0.79-2.40) | 0.26 |  |  |  |  | 0.75 (0.39-1.44) | 0.39 |  |  |  |  | 1.35 (0.63-2.87) | 0.44 |  |  |  |
| AML, acute myeloid leukemia; BMT, bone marrow transplantation; CB, cord blood; CBT, cord blood transplantation; CNI, calcineurin inhibitor; DRI, disease risk index; ECOG PS, Eastern Cooperative Oncology Group Performance Status Scale; HCT-CI, Hematopoietic Cell Transplantation-Comorbidity Index; MAC, Myeloablative conditioning; MMRD, HLA-mismatched related donor; MMUD, HLA-mismatched unrelated donor; MRD, HLA-matched related donor; MTX, methotrexate; MUD, HLA-matched unrelated donor; PBSCT, peripheral blood stem cell transplantation; and RIC, reduced intensity conditioning. | | | | | | | | | | | | | | | | | | |
