# Supplementary material for: Prognostic impact of effusion in multiple body cavities after allogeneic hematopoietic stem cell transplantation
Source: Int J Hematol. 2025 Mar 3;121(6):833–47. doi: 10.1007/s12185-025-03949-7 (PMC12106147; doi:10.1007/s12185-025-03949-7)
Supplement: Supplementary file 3 — Supplementary file3 (DOCX 18 KB) [file 12185_2025_3949_MOESM3_ESM.docx]

| **Supplementary Table 3. Patient characteristics stratified by revised DRI.** *N*=175, excluding three patients with their DRI not confirmed whether low/intermediate or high/very high. | | | | |
| --- | --- | --- | --- | --- |
|  |  | Low/intermediate (*n*=104) | High/very high (*n*=71) | *P* values |
| Age at transplantation (yo) - median [range] | | 48.3 [17.1 - 70.6] | 54.6 [19.7 - 69.3] | **0.005** |
| HCT-CI - median [range] | | 0 [0 - 6] | 1 [0 - 6] | **0.03** |
| ECOG PS - median [range] | | 0 [0 - 3] | 1 [0 - 3] | 0.08 |
| Cytotoxic chemotherapy (cycles)* - median [range] | | 3 [0 - 20] | 3 [0 - 12] | 0.68 |
| Time from diagnosis to transplantation (days) - median [range] | | 204 [45 - 8,866] | 178 [18 - 4,707] | 0.05 |
| Albumin at transplantation (g/dL) - median [range] | | 3.7 [2.2 - 4.7] | 3.6 [2.1 - 4.8] | 0.69 |
| EASIX pre-transplantation - median [range] | | 1.0 [0.0 - 39.8] | 3.0 [0.4 - 111.4] | **<0.001** |
| Conditioning - no. (%) | |  |  | **0.03** |
|  | MAC | 71 (68.3) | 37 (52.1) |  |
|  | RIC | 33 (31.7) | 34 (47.9) |  |
| Time to first effusion (days)† - median [range] | | 25.5 [5.0 - 2,950.0] | 22.0 [2.0 - 1,524.0] | 0.44 |
| ES - no. (%) ‡ | | 5 (5.1) | 3 (5.5) | >0.99 |
| Grade 2-4 acute GVHD - no. (%) ‡ | | 41 (41.8) | 31 (56.4) | 0.08 |
| TMA - no. (%) ‡ | | 5 (5.1) | 2 (3.6) | >0.99 |
| SOS - no. (%) ‡ | | 5 (5.1) | 1 (1.8) | 0.42 |
| Bacterial infection - no. (%) ‡ | | 64 (65.3) | 36 (65.5) | 0.99 |
| CMV infection - no. (%) ‡ | | 6 (6.1) | 3 (5.5) | >0.99 |
| CMV, cytomegalovirus; ECOG PS, Eastern Cooperative Oncology Group Performance Status Scale; GVHD, graft-versus-host disease; HCT-CI, Hematopoietic Cell Transplantation-Comorbidity Index; SOS, sinusoidal obstruction syndrome; and TMA, thrombotic microangiopathy. * Excluding one (RDRI low/int) and two (RDRI high/very high) patients with insufficient clinical records available. † Excluding 42 (RDRI low/int) and 12 (RDRI high/very high) patients without effusions. ‡ Number and proportion of patients with specific complications by day 100, out of 98 (RDRI low/int) and 55 (RDRI high/very high) who survived until day 100 are shown. | | | | |
